# Co-design for technology in paediatric therapy: Developing an augmented reality intervention for children with developmental coordination disorder

**DOI:** 10.1177/20556683241266780

**Published:** 2024-08-08

**Authors:** Ellana Welsby, David Hobbs, Brenton Hordacre, Emily Ward, Susan Hillier

**Affiliations:** 1 Allied Health & Human Performance, Innovation, IMPlementation and Clinical Translation (IIMPACT) in Health, 64772 University of South Australia, Adelaide, SA, Australia; 2 College of Science and Engineering, Medical Device Research Institute, 117627 Flinders University, Tonsley, Adelaide, SA, Australia

**Keywords:** Co-design, assistive technology, paediatric therapy, developmental coordination disorder, therapeutic intervention

## Abstract

**Background:** Children with developmental coordination disorder (DCD) have difficulty learning and performing movements, often requiring increased feedback. Technology may be useful for delivering augmented feedback. Co-design methodology for developing therapeutic interventions has become popular in healthcare, including for technology in rehabilitation. However, there are limited guidelines on how to use co-design methodology in healthcare. **Methods:** We applied three key principles, (1) Understanding, (2) Exploring, and (3) Materialising, to inform a co-design process. End-user participants included paediatric clinicians, young learners, their caregivers, and the research team, who shared their expertise and lived experience to inform the creation of a novel system. **Results:** A team of end-users designed and developed an augmented reality intervention prototype for practicing motor skills aimed at children with DCD using a generative co-design process. From understanding the unmet needs, we explored and then materialised a series of games using bespoke technology solutions. **Conclusion:** The use of a co-design process was beneficial in engaging end-users as the experts of their experience in the creation of a novel augmented reality intervention prototype aimed for children with DCD. The co-design process was successful in facilitating a prototype that meets consumer needs, and ensured all end-user voices were heard.

## Introduction

It is well known that rapid uptake of high-quality interventions, clinical procedures and technologies is needed to attain the best possible healthcare outcomes.^1^ However, a large gap exists between research outcomes and their translation into clinical and healthcare practice. A key contributor to low uptake of research into clinical practice is that the relevance of key studies and research questions to health practitioners and end-users is poor.^2^ One way that researchers and funding bodies have been trying to improve clinical translation is by employing a co-design method. Co-design is the process of involving key stakeholder groups at all stages of a development process to ensure that the voices of the intended end-user(s) are heard.^3,4^ Due to the emphasis on collaboration, co-design has become popular to ensure end-user needs are met and that research, a product or service design has value to end-users to achieve more usable products, increase uptake, and improve efficiency of healthcare practices.^5–7^ Despite its importance, limited opportunities are typically provided to collaborate with end-users in the creation and design of health services.^5,8,9^ Most end-user involvement is sought as feedback on an already developed product, limiting the influence of lived experience and end-user needs, and therefore, resulting in low uptake of the intended product.^5^ The co-design framework aims to address this gap by enabling high-level engagement for all end-users at the creation, design, and development stages, ensuring the product is relevant and appropriate for the intended audience.^8,10^ The creation of a product, service and/or policy that is derived from end-user voices, understood by all stakeholders, tailored to the population, enriched by knowledge and expertise, and fits an identified unmet need, is best placed to drive change in the healthcare system.^5,8,10,11^

The co-design approach is also compatible with the main aims of The International Classification of Functioning, Disability and Health: Children and Youth (ICF-CY) framework. The ICF-CY was created from the original version of The International Classification of Functioning, Disability and Health (ICF) to provide a global conceptual framework for identifying disability and developmental issues experienced by children and youth.^12^ The framework emphasises the importance of promoting participation regardless of disability, providing opportunity for individuals to shape their environment and experiences, managing environmental affordances and barriers, and facilitating collaboration.^12^ Co-design methodology closely aligns with and is best placed to address all of these aims by empowering individuals with lived experience through active voice and opportunity in the development of products or services that affect them.

One area evolving rapidly is the use of technology within therapy. As technology continues to evolve and is embedded in more aspects of our daily lives, it has become particularly important in healthcare as an innovative way to deliver education and therapy. For example, when rehabilitating movement, the opportunity for enhanced (augmented) feedback modalities has changed the way we can help children and adults, with or without movement difficulties, engage in rehabilitation or to learn new skills.^13–17^ Developmental coordination disorder (DCD) is one condition that may benefit from technological interventions, particularly those that offer increased practice, augmented feedback, and environmental accessibility. Children with DCD typically experience difficulties with motor coordination, performing motor skills below what is expected for their age, and they have difficulty learning and refining motor skills.^18,19^ Research suggests they may need support through enhanced feedback to recognise error signals during motor learning, and may need better ways to evaluate and refine their performance.^20^ Feedback is a crucial element to the success of learning a motor skill, facilitating the update of our stored motor plans through feedforward internal models of movement.^21,22^ Feedback can be delivered before, during or after the completion of a motor task and can be provided via internal (within the body), or external (outside the body) modalities. Evidence suggests technology aimed at motor performance and motor learning may be well placed to deliver customisable levels of feedback, including for children with DCD, through goal-directed and performance-based interventions.^23^

Currently, commercially available gaming consoles are being used to deliver therapeutic interventions. For example, the Microsoft Wii, Nintendo switch and Xbox Kinect, are being used to help children learn new motor (hand-eye coordination, balance) and cognitive skills (decision making, switching attention).^24–27^ It is important to note that while most of the consoles and games available seem to be effective as a therapeutic intervention, they have not been created with therapeutic intent, rather, for commercial use. Co-design of a specific therapeutic gaming intervention by experts and end-users may produce further positive outcomes for the intended user. To our knowledge, a customised intervention using technology for motor skill learning in children with DCD, using the co-design method, has not been created.

This paper describes the process that was used to co-design and develop an augmented reality intervention aimed for children with DCD to support their motor skill development. The aim of this project was to design a system that could be used as an accessible and engaging intervention tool, using a game-based approach, aimed for children with DCD that promotes a novel way of practicing a motor task, and that can be used as a therapeutic tool by paediatric therapists in multiple environments.

## Methods

### Co-design participants

For the purpose of this project, relevant end-users were identified as including clinicians who would potentially use the intervention, researchers as part of the research team facilitating the project, parents and/or caregivers of children with or without motor difficulties, and the children themselves. Clinicians were required to have worked clinically with children with DCD in the last 5 years and have experience with children with motor difficulties. There were no minimum years of experience required, however, the clinicians who were approached had a minimum of 5 years’ experience. Participants within the parent and/or caregiver and young learner groups were not required to have any experience with gaming for rehabilitation. The research team were chosen for their combined experience in serious gaming, rehabilitation and therapeutic rehabilitation for children. A software developer was also employed to develop the system and was involved in the co-design process as a key part of the team. All participants of the co-design team brought a unique perspective to the design of the prototype.

Paediatric allied health clinicians were selected via a convenience sample and invited to take part in the study. Clinicians were selected based on their clinical and/or research experience working with children with DCD and/or motor difficulties. Eight paediatric allied health clinicians were contacted to participate in this study. Disciplines included occupational therapists, physiotherapists, and a psychologist.

Due to COVID-19 restrictions children and their parents/caregivers were unable to be recruited for the initial phases of the co-design process. Children were recruited to the young learner’s group to evaluate and test the system when it was nearing the final stages of development. Four typically developing children (between 8 and 12 years of age), and one child (10 years of age) who was identified as ‘at risk’ of motor delay (≤16% Movement Assessment Battery for Children, 2nd Edition), were recruited to test and provide feedback on the system in its penultimate development stage. Each participant was accompanied by their parent(s), who also provided informal feedback on their child’s engagement with the prototype system. Informal qualitative feedback on the use of the system for their children was captured during the sessions. Data on the parent perspective was also captured within the clinician and researcher groups, as most were parents of young children. One member of the research team also participated as a parent of a child with DCD.

The research team consisted of five researchers with various experience. Specifically, one occupational therapist, two physiotherapy senior researchers (PhD), one rehabilitation and biomedical engineer researcher (PhD), and one professor in neuroscience and rehabilitation. Combined knowledge and expertise covered the areas of neurorehabilitation, child development and paediatric conditions (with extensive experience with motor conditions), serious gaming, product design, and emerging technologies. Two of the included participants in this study are also authors of this paper, to continue the cycle of end-user engagement, from concept to implementation and dissemination of the design process, and ensure all voices are being represented.

Ethical approval was obtained from the University of South Australia Human Research Committee (ID: 202959). All participants provided written informed consent prior to their participation in accordance with the World Medical Association Declaration of Helsinki.

### Co-design approach

This study forms the first phase of the Virtual Reality Clinical Outcomes Research Export Model (VR-CORE) which is being used and adapted as a best practice framework to inform the creation of a co-designed augmented reality intervention.^28^ The first phase, VR-1, is intended to focus on content development with end-user’s to inform the creation of the technology to be designed.^28^ Co-design methodology has been chosen to inform this process.

The importance and benefits of inclusion of healthcare practitioners, recipients of treatment and service providers in healthcare product design has been well demonstrated.^8,29–31^ However, co-design in the literature is inconsistent, and therefore there was no one guideline to follow on how to run the co-design process.^9^ To accomplish the project aims, a design thinking approach, adapted from Hobbs et al. (2019), was chosen to guide the co-design process, utilising three key principles, (1) Understanding the problem from detailed research and end-user concerns, (2) Exploring the needs of end-user groups to define the problem and create solutions; and (3) Materialising solutions through prototypes, testing and evaluation.^30^

### Co-design process

#### Understanding the problem

To understand the problem, in the initial stages of the project the research team conducted extensive inquiry into DCD which included current therapy and supports available for children with DCD, and future directions and recommendations; motor learning in typically developing children and children with DCD; and parent perspectives of interventions for children with DCD. The existing literature was supportive of providing visual and auditory feedback rather than only enhancing haptic or interoceptive feedback.^23^ Additionally, there appeared to be no convincing evidence as to the importance of timing of the feedback for this population.^23^ Next, research into the needs of end-users was conducted with paediatric clinicians via questionnaires that helped define the problem and brainstorm potential solutions. From the initial inquiry it was decided the new intervention would be designed using augmented reality technology that would target the development of motor skills using a game-based approach for children with DCD. The research team chose the Microsoft Kinect Azure DK due to its advanced body tracking capability to form the foundation of the intervention. Therefore, it is important to note that there was a broad idea of what the intervention might involve in terms of providing feedback through augmented reality to facilitate motor learning, however, there was no preconception of what the intervention would necessarily include or look like.

##### Questionnaires

**Figure 1.**
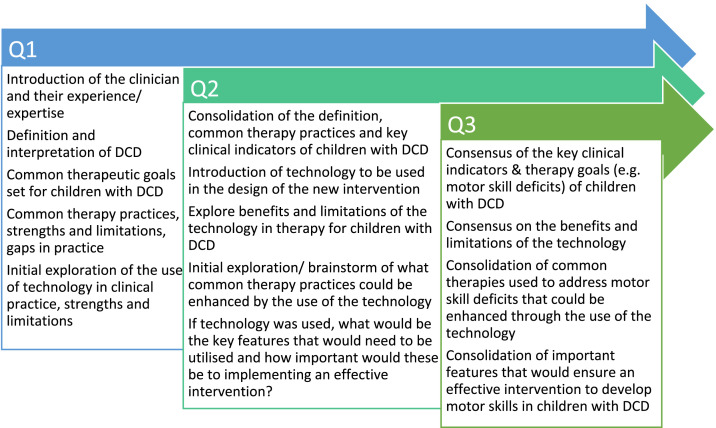
Identify and understand the problem: Description of the questionnaire rounds.

Three questionnaire rounds were delivered 1 week apart to gather information about the participants, introduce ideas and potential technology, and begin the brainstorming process. Each subsequent questionnaire was built on from the previous questionnaire’s answers, and used to disseminate collective answers, and explore ideas and concepts each participant added. See Figure 1 for a detailed description of the questionnaire rounds.

Questionnaire 1 focused on the clinician and their experience with children with DCD and how they define, interpret, and treat children with DCD. The clinician group was asked to reflect on their clinical practice and knowledge of DCD, and strengths and limitations of common assessments and interventions they use. Questionnaire 2 was used to introduce the potential technology that could be used and to explore the benefits and challenges to using technology for therapy in practice. This questionnaire also included videos of the working technology and a list of features the technology could provide. The clinicians were then asked to rate the importance of each feature to delivering an effective intervention. Questionnaire 2 ended with a brainstorming activity, where clinicians were prompted to list the types of interventions they have previously used with children with motor difficulties, such as with DCD, that could be delivered using technology. Questionnaire 3 was developed from the answers given in Questionnaire 2 with the aim of selecting the top features, motor skills and activity ideas to bring to the workshop phase. This questionnaire also highlighted and rated key features expressed by participants in the previous questionnaire. Answers from all three questionnaires were collated and prioritised to form the activities for the following workshops.

### Explore needs, define the problem, create solutions

#### Workshops

A total of four workshop rounds were delivered to explore and define the need, and to create the initial design. The first two workshops were delivered face-to-face for 1 hour, 2 weeks apart; and the third and fourth workshops were delivered in the form of an online document as it was not possible to meet in person at that time due to COVID-19 restrictions. Along with each of the clinicians, a software developer also attended the workshops to answer any questions related to technology capability and functionality and to gain an understanding of the product design from an end-user perspective.

Workshops were facilitated by the lead researcher and structured to provide opportunity for all workshop members to share their various experiences and ideas. Workshop one was focused on introductory activities to create a cohesive and open space to share ideas between the workshop members. Discussion around the concepts and ideas from the questionnaire rounds were facilitated, including ideas about meeting children and parent/caregiver needs, from both a clinician perspective and personal experience as parents. The Microsoft Kinect Azure DK camera was also demonstrated and used by the clinician group, which prompted discussions about which motor skills the technology would be most suited to, considering the technology’s strengths and weaknesses. Clinicians were encouraged to brainstorm, and mind map potential motor skills as well as how to ‘gamify’ the learning and practice of the motor skill.

The second workshop aimed to select five key motor skills identified by the clinician group as the most important motor skills for children with DCD and begin to ‘gamify’ them. Once the key motor skills were identified, the clinicians were presented with options of delivery that the technology could support. Subsequently, they were involved in discussions and mind mapping activities to explore and consolidate essential features and settings that were identified as most important to facilitate accurate motor learning in each of the chosen games. The group was then directed to brainstorming ‘themes’ and ‘scenes’ for each identified motor skill. The clinicians were also asked to vote on the structure and method of delivery to teach the motor skill games. Additionally, the members were encouraged to discuss the strengths and limitations of the use of the system in different environments (i.e., clinic, school, home), and how the system would increase motivation, engagement, and socialisation/interaction.

At the end of each workshop round, an email was sent to all participants summarising the discussions and outcome of the workshop. Participants were then provided with an opportunity to give feedback on the outcomes of the workshop and seek clarification if it was required.

The third and fourth workshops were unable to take place in person, so they were adapted to be facilitated via an online document. A storyboard was created detailing each of the five chosen motor skills with their corresponding design and themes, plus important features, including skill focus, body position, visual feedback, auditory/narration feedback, and other types of feedback. Each skill was written in ‘steps’ of performing the motor skill (e.g. how to perform a jump) that would reflect the target skill and age range of children. Clinicians were asked to provide comments on all aspects of the motor skills, themes, and game-based feedback from their clinical experience. Each clinician filled out their storyboard separately and once all feedback was received, answers were collated into a single document. The collated answers were then sent back out to all clinicians to review and refine.

Once the storyboard had been sent to the clinicians for two workshop rounds, the document was then sent for evaluation to the research team who provided further expertise and clinical experience to evaluate and define any missing criteria. This was completed through two evaluation rounds that contributed to the co-design process. This was the final step of the explore phase.

### Materialise and prototype

#### Storyboards

Following the workshop rounds and evaluation of the initial storyboards, the motor skills, themes, and features that were selected by the clinician and researcher expert groups were finalised in a storyboard to provide to the software developer. Two different formats of storyboards were produced. The first was a detailed written description of all tasks, feedback, and scenes and the second was a hand drawn storyboard to provide visual participant perspectives when playing the games and step-by-step instructions for each of the movements. These were handed to the software developer to materialise the first prototype of the game.

#### System development and evaluation

The next phase of the co-design process was the development of the system that would form the intervention. This phase included multiple prototypes that were tested and refined according to the storyboards created by the expert groups. The lead researcher worked in collaboration with the software developer through multiple stages of testing and refining of the developing intervention.

#### Testing and evaluation with young learners and parents/caregivers

**Figure 2.**
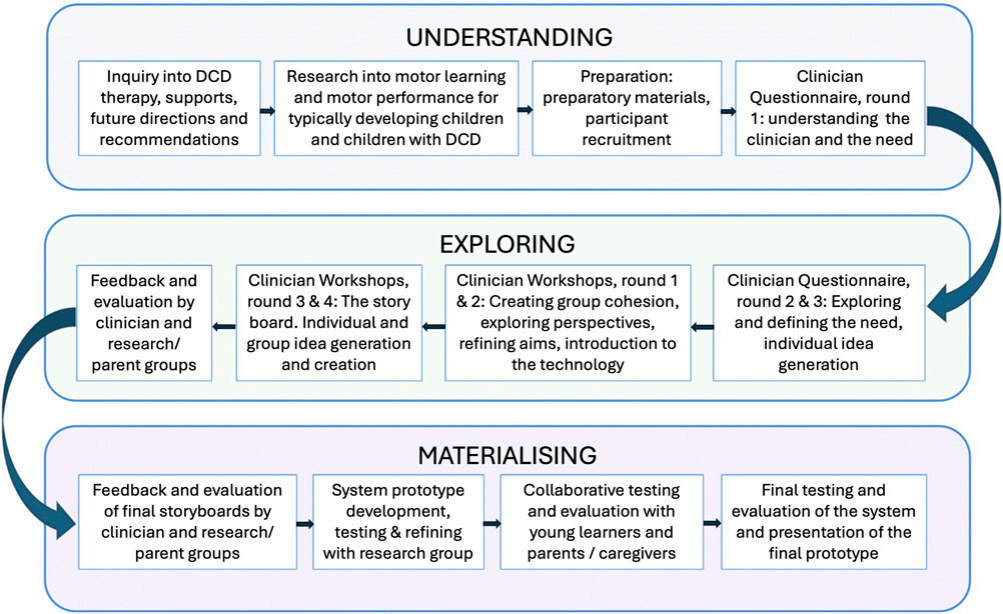
The co-design process.

Once a final prototype was developed, to complete the co-design phase, the new system was tested and evaluated with five children, with and without motor difficulties, who provided feedback and suggested adaptations. Feedback was collected via questionnaires that included open ended questions and a Smiley Face 5-point Likert Scale. Parent perspectives were also collected via informal discussions throughout the sessions. Following this, the prototype went through two more testing phases with the lead researcher and software developer to fix any inconsistencies and the system was finalised for future testing. The co-design process is explained in full in Figure 2.

## Results

Through the co-design process, an augmented reality motor skill intervention prototype was created to help improve motor skill performance for children with DCD. The intervention was designed to go into the next phase of testing, which was the feasibility, usability, and acceptability of the intervention within healthcare settings with children with DCD.

### Expertise and role of clinicians

A total of five clinicians formed the expert group for the co-design process, including three occupational therapists and two physiotherapists who specialise in paediatric therapy. Two of the clinicians were practicing in private paediatric clinics with between three and 6-years’ experience, and stated they would each see on average between 1 and 5 children with DCD per week. The remaining three participants were clinicians who previously worked in paediatric practice and now practice in paediatric research, all with 15+ years of clinical experience. The initial questionnaires revealed that each of the clinicians had similar definitions and thoughts about therapy for children with DCD. All five clinicians thought technology was an effective intervention for children with movement disorders and specifically for DCD. Common opinions expressed were that ‘*children are attracted to technology*’ and that ‘*it can be used effectively as a motivational tool*’ to engage with therapy. Additionally, it was expressed that technology can be used to ‘*create controlled environments and experiences that can be adapted/refined to suit the individual*’ and that it is able to provide a *“safe space”* for children with motor difficulties to explore their environments ‘*without putting the child at risk in the “real world”*’. While all clinicians’ agreed technology was effective for interventions for children with motor difficulties, only three of the five clinicians had used it with children in therapy. Interestingly, the two clinicians who had not used technology were private practice clinicians not involved in research.

#### Input from the research team

The research team provided valuable background information through a systematic review of the literature providing the best available evidence to help understand the current evidence on feedback modalities, including through technology, for motor learning in children with DCD, and added further clinical and research experience to aid in the co-design process.^23^ The co-design process this study followed, involved an ongoing cycle between the clinician group, the researchers, young learners and the software developer to ensure a cohesive structure that placed clinicians and children (as the end-users) at the centre of the design. The research team helped to ensure no steps were missed in the co-design process and provided secondary evaluation and adaption by adding further clinical expertise, lived experience as parents, and knowledge to the design of the intervention.

#### Clinician group: Selected motor skills & themes

During the questionnaire rounds the clinician group were asked to list the primary motor skills they treat in their clinical practice for children with DCD. From here, these were collated and refined to create a list of the top gross motor skills/activities and top fine motor skills/activities treated within the clinic. The top gross motor skills/activities identified were run, hop, vertical jump, catch and kick. The top fine motor skills/activities were grasping and manipulating objects and scissor skills. For the full list of skills/activities identified see Table 1.

**Table 1.** The top rated gross and fine motor skills/activities as voted by clinicians following the questionnaire rounds.

Gross motor skills/activities	*n*	Fine motor skills/activities	*n*
Run	4	Grasp	4
Hop	4	Scissor skills	4
Vertical jump	4	Manipulating objects	4
Catch	4	Handwriting	2
Kick	4	Drawing	1
Stationary dribble	1	Construction	1
Slide	1		
Gallop	1		
Striking a stationary ball	1		

Ideas about motor skills to target changed quickly with the demonstration and practice with the technology and this facilitated more detailed and in-depth ideas around how the technology could be best used. It was noted the technology could be used within a ‘bottom-up approach’ where the foundation skills required to perform activities of daily living are practiced discretely through a fun and engaging game. For example, practicing postural control to assist with handwriting, hand eye coordination to assist with dressing tasks, and balance training to assist with sport activities such as riding a bike. While discussions often revolved around ‘what’ the intervention would include, another important question was ‘how’ the technology would achieve its goal of teaching and practicing motor skills.

By the end of the questionnaires and round two of the workshops, the clinician group had cohesively discussed and made final decisions on 5 key motor skill games, structure, and themes for each game, and decided on the ‘most important’ features of the technology to the most appropriate intervention for children with motor difficulties.

#### Clinician and researcher groups: Important features

**Figure 3.**
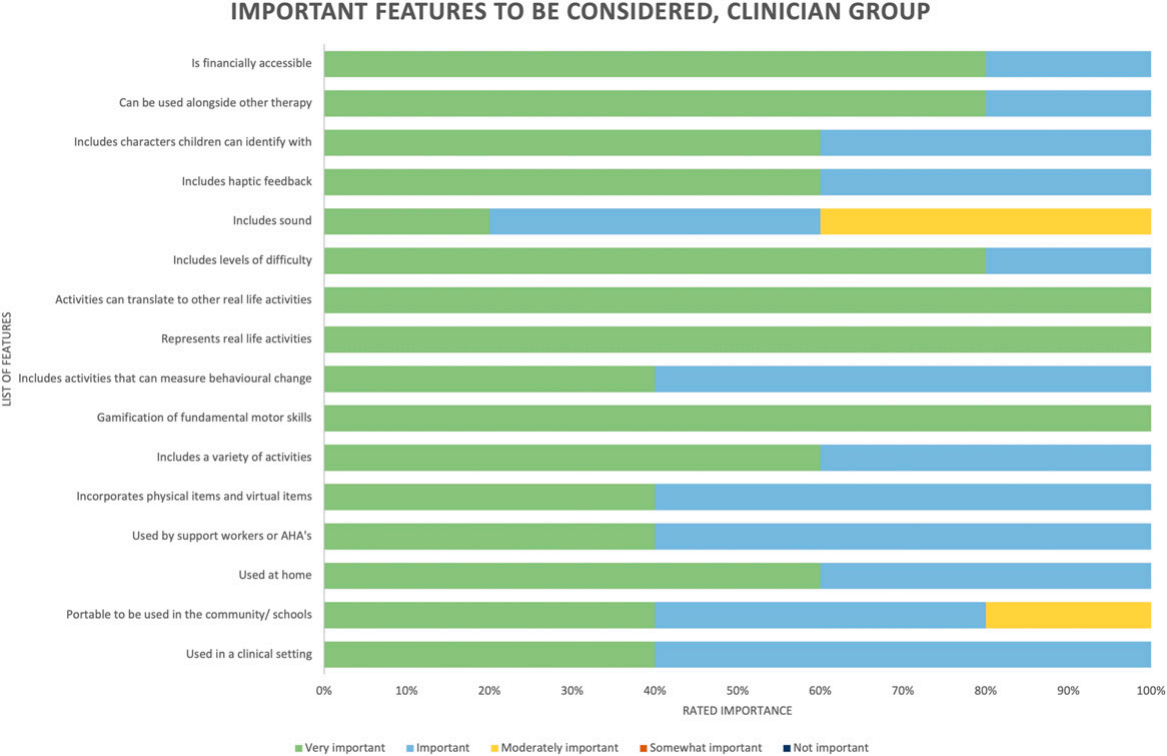
List of features rated by importance by the clinician group.

An important conversation during the co-design phase was what features were most important for the technology to include to facilitate motor skill practice. See Figure 3 for the full list of features as voted by clinicians. One of the most obvious criteria was that the technology needed to be accurate with capturing movement to ensure appropriate, and ‘*real-time’* visual feedback was given to facilitate motor learning and practice. Other key features that were voted as being ‘important’ included that the intervention can be used in a range of settings or environments and can be used by multiple health professionals. It was voted as ‘very important’ that the intervention included a variety of activities and themes to keep the intervention engaging and motivating, that the activities chosen were representative of *‘real-life’* activities and that they could be easily translated to other activities of daily living.

*Levels of difficulty* were also discussed as one of the ‘most important’ features for the game to include. Levels of difficulty were defined as varying degrees of difficulty and challenge that players can choose from when engaging with the game. Multiple discussions by the clinician group concluded that the intervention must be able to be tailored to the individual to match the individual’s performance level and preferences to create the ‘*just right challenge’* for the participant.

Along with accurate visual feedback, auditory feedback was also highlighted as an ‘important’ feature to facilitate ease of use and understanding for all ages and skill levels. Incorporating positive ecologically valid sounds for the ‘*reinforcement of correct movements*’ was also ‘very important’, as well as positive comments that reinforced correct movements or that would encourage a participant to try the movement again if done incorrectly. Interestingly, the clinician group did not consider sounds for incorrect movement as important, rather a visual cue was suggested as being more effective, for example not matching the shape outlined on the screen. Music to accompany the intervention was also voted to be an ‘important’ feature to make the intervention feel ‘*game like*’ and enticing for children.

**Figure 4.**
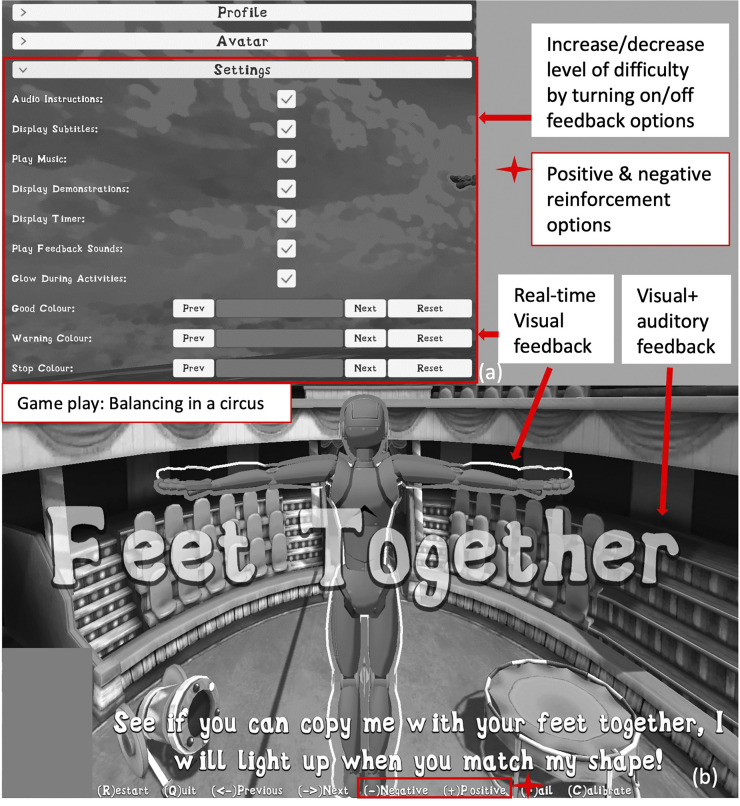
(a) Displays the feedback options incorporated in the game that the individual user and/or the operator can select to increase or decrease difficulty. (b) Demonstrating the user experience within a game level, showing the various feedback elements on display.

Finally, the importance of haptic feedback was discussed. Haptic feedback refers to the tactile vibrations or sensations while playing a game or moving within the natural environment. Haptic feedback is usually delivered via a controller when gaming and by the body’s sensory system in the natural environment. While haptic feedback may be an important part of motor learning, the clinician group was divided as to whether the intervention needed to add or enhance the haptic feedback the user receives beyond what they would experience intrinsically with the technology. After lengthy group discussion it was decided that enhancing haptic feedback with this specific intervention was not a key feature, although could be an option for future developments. The clinician group also identified important features were that the intervention would be able to be used alongside traditional therapy methods and that it is financially accessible. To see how these important features were represented in the prototype for testing and evaluation see Figure 4.

#### Young learner’s evaluation and feedback

**Figure 5.**
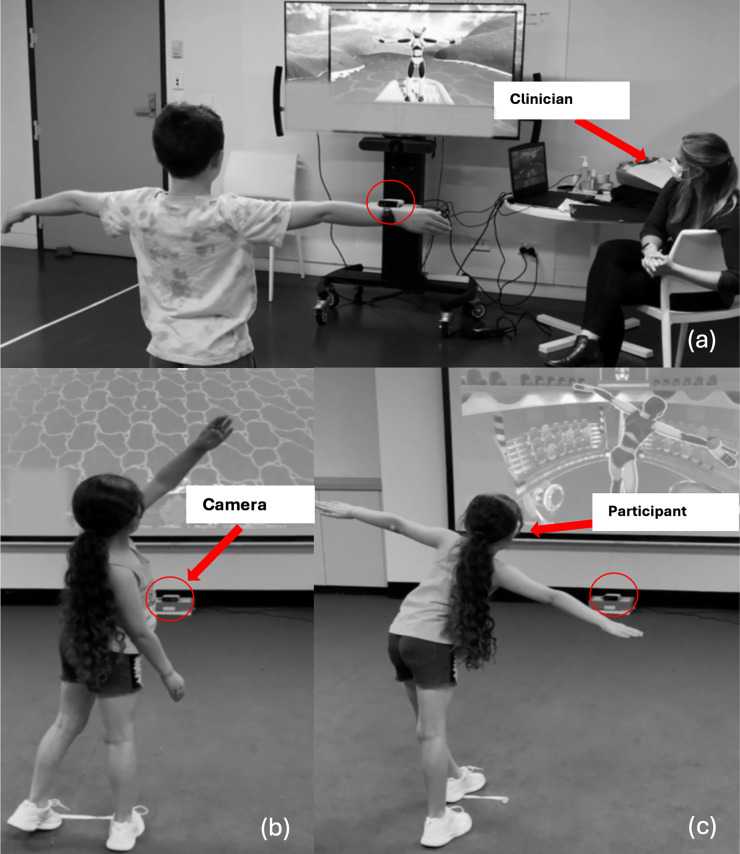
Early trialling of the system with young learners in the testing and evaluation phase of the project, using an early version of the system. (a) shows a young learner using the system with a clinician in a clinic space, while (b) and (c) show a young learner interacting with the system to complete different modes in a school classroom. The Microsoft Kinect Azure DK camera capturing movements can also be seen (circled in each photo).

The overall experience of the young learners who tested the intervention at the prototype stage was positive. The young learners rated the enjoyment of using the technology an average of 4/5 (range from 3/5 to 5/5) on the 5-point Smiley Face Likert scale, stating the intervention ‘*was fun*’. Specifically, the young learners identified that they enjoyed that the game involved ‘*physical activity*’ and ‘*screen time*’ and stated that their friends would enjoy playing too. On average, 3/5 of the young learners rated they would ‘*absolutely*’ use the system at home (range from 2/5 to 5/5) and that ‘*yes*’, they would use the system at school with friends (range from 4/5 to 5/5) on the 5-point Smiley Face Likert scale. A mixed response was given when asked if the young learners liked the themes/animations in the game, with 3 reporting they ‘*absolutely*’ liked the themes and the remaining 2 reporting they ‘*maybe*’ liked the themes. Some suggestions for improvement were given, including that some of the objects within the games could be changed from ‘realistic’ images to more ‘cartoon’ images, and that more music could be added to make the game ‘*more exciting*’. See Figure 5 for a demonstration of the test and evaluation sessions.

#### Parent perspectives

Parent feedback was collected informally through subjective reports to the lead researcher during the testing and evaluation sessions with their child and via clinician and researcher perspectives throughout the ‘understanding’, ‘exploring’ and ‘materialising’ phases. Exploring the literature of parent perspectives for intervention for children with DCD suggests parent-identified needs are not being met to help their children develop and participate in the community and at home.^32,33^ Additionally, it is reported many parents and/or caregivers would like more supports and education at school to support their children’s needs.^32,33^ These findings were confirmed through our participant groups. Parents of the children who were involved in testing and evaluation reported they liked the system because it provided a *‘fun’* and *‘engaging’* way for their children to learn, *‘without knowing they are learning’*. One parent noted it provided a way for their *‘child to practice the skills they need help with, without the pressure of being in therapy’* and that this is important for helping their child to *‘not feel different or segregated’* from their peers. The parent representative from the researcher group who has a child with DCD, noted the system provided a ‘*safe’* way for therapy to occur in ‘*any environment’*, including at school, home or in the clinic, with *‘the use of technology’*. They also noted that the system *‘was easy to use from a parent perspective’* and is *‘intuitive’,* so that it *‘does not need to be driven by the parent’*, for the practice of motor skills to occur. These concepts were also noted by the clinician group as ‘very important’ when asked about common feedback from parents and/or caregivers in their clinical experience.

## Discussion

The aim of this co-design project was to design a new intervention using technology aimed for children with DCD. Partnering with paediatric allied health clinicians, researchers in paediatric movement disorders, and young learners within the target age group, through a co-design framework, facilitated the design and prototype of a novel game-based motor skill intervention aimed for children with DCD. The final prototype reflected the identified gaps in clinical practice as recognised by researchers, clinicians, and parents and/or caregivers, facilitated knowledge translation between these groups, and created a system and intervention that met the wants of both parents and/or caregivers, clinicians, and the young learners as participants of therapy. It is hoped this game-based approach will assist children experiencing motor difficulties, such as DCD, to engage and benefit from motor skill practice where the individual learner is able to find their own level of challenge. The next step of this project is to continue the testing and evaluation of the feasibility, usability, and effectiveness of the intervention with these children in different settings.

### Key findings

Technology as a provision of therapy for the paediatric population was at the centre of the design and was identified by all key groups as an important part of emerging healthcare practices and a *‘want’* for clinical practice. The power of co-design is to ensure a product meets the needs and requirements of all end-users.^5–7^ This project supported the opportunity for the transfer of knowledge and experience between researchers, clinicians, parents and/or caregivers and children that ultimately shaped a system that met multiple end-user needs. Through the co-design framework we were able to facilitate conversation and discussion around the similarities and differences in experiences between groups, which moulded the final prototype. Ideas were shaped, reformed and/or strengthened through the co-design framework.

Accuracy of movement was the most important feature for all groups, along with incorporating the gamification of realistic activities into the intervention. However, the motivation for these features differed. For the clinician and researcher groups, accuracy of movement represented precise feedback that is important for clinical and behavioural outcomes, and the gamification of realistic activities was important for the transfer of learnt skills to other activities of daily living. For the young learner’s group, the accuracy of movement was key in creating immersion within the game, and to facilitate ‘gameplay’. Additionally, the gamification of realistic activities was important to mimic their favourite games they play with others, highlighting the importance of socialisation. Incorporating ‘*levels of difficulty*’ was also identified as ‘very important’ for the clinician and researcher groups to ensure individual learners can find their own level of challenge. This was also reflected by the young learners group who rated their experience with the intervention positively as each experience was customised to their performance level. Parents of the young learners also noted they liked the intervention because it was ‘fun’ and ‘engaging’ for their child and provided a ‘safe’ way to learn. The visuals, animations and sound/music within the game were very important to the young learners to create the sense of a game ‘story’, however, for the clinicians this was only voted as a moderately important feature. Clinicians felt the most important features were to facilitate feedback on a movement, rather than the creation of a storyline or flow of animation.

The parent perspective added another viewpoint, highlighting the importance of watching their children *‘have fun with therapy’* and for their children to not feel *‘different or segregated’* from their peers. Parents also highlighted the importance of the transferability of the system to different environments, and the *‘safe’* experience it provides, including within the user’s environment, but also that it provides a socially and emotionally *‘safe’* space for their children to engage in the practice of motor skills. These similarities and differences highlight the importance of co-design to meet the needs of all end-users to create the most effective product.^5–7^

### Strengths and limitations of the study

Although co-design is increasingly recognised as an important method to improve clinical translation and to create products that suit end-user wants and needs, published studies lack detailed methods, limiting the translation of high-quality co-design methods into practice. To address this gap, detailed methods of the co-design process that was based upon a conceptual framework are provided in this study to increase transparency and promote the replication of an effective co-design research method.

Using a co-design framework within healthcare empowers included groups by incorporating the lived experiences and voices of end-users in research to ensure an intervention is tailored to the requirements of users, and therefore, increases the likelihood of adoption and integration of the product.^8^ One of the strengths of this study was the emphasis on the inclusion of end-users from the project’s inception to creation of the prototype. Co-design methodology requires the research team to remove any preconceived ideas or thoughts to avoid bias within the process. It is crucial that the identified gap is conceived through a collaborative process between all involved end-users. In this study, emphasis was placed on building rapport with all members of the study groups and using different methods of idea generation to ensure ideas, needs and wants of all end-users were heard and met.^34^ Within the clinician and researcher expert groups, ‘group effect’ bias was minimised by facilitating idea generation and conceptualisation activities in both a group and individual settings to ensure all participant voices were heard.

A limitation of the process within this study was that children with and without DCD and their parents and/or caregivers were unable to be involved in the initial conception and exploration of the identified need due to COVID-19 restrictions. Additionally, we were unable to recruit a larger number of children diagnosed with DCD. To ensure the young learners’ group were able to be involved in the co-design process, they were given opportunity to provide design feedback as early as possible in the materialise phase of this project and were prompted with similar design questions as the clinician and researcher groups. In future projects, it is recommended all end-user groups are given the opportunity to be involved from conception to implementation of the product to ensure all needs and wants of user groups are met and that all information is collected via a formal process to ensure no information or feedback is missed.

We hope that the implementation of individual sessions may have minimised a ‘group-effect’ bias by allowing those who may find it difficult in group situations to offer their opinion to have a voice. Future co-design projects may consider number and diversity of included groups as well as creating affording environments and activities to facilitate innovative ideas and creation.

### Future directions

This study presents the co-design method and findings for the first prototype of an augmented reality intervention aimed for children with DCD. To continue the design process, the intervention will continue to be developed, tested, and evaluated with the target paediatric group to determine the feasibility, usability, and acceptability of the system for the intended population. To ensure the system continues to accurately meet end-user needs as it develops, end-user engagement and involvement in all steps of the design will be a priority to ensure the translation of the intervention into healthcare.

## Conclusion

Using a co-design approach to develop a technology intervention aimed for children with DCD was a beneficial technique for the development of the system prototype. Through the design process, the clinician and researcher groups were able to learn about the potential role of technology in paediatric therapy and were able to develop ownership and engagement of the technological advancement in healthcare to inform their practice. Children and their parents and/or caregivers who would also benefit from the intended intervention, were empowered to be experts of their own experience and involved in an innovative process of the development of future technology. Based on the co-design experience, we recommend healthcare projects and innovations use a co-design framework, where end-users are involved in the project from inception to implementation to ensure a shared understanding of the unmet need between researchers and end-users and to ensure translation to healthcare settings. It is hoped the prototype will continue to grow and develop with end-users to inform the final desired product. Co-design is a useful and beneficial way to engage end-users as the experts of their experience and facilitate clinical translation and adoption in healthcare settings.
